# Supplementary figures and images for: Rhesus negative males have an enhanced IFNγ-mediated immune response to influenza A virus
Source: Genes Immun. 2022 Apr 15;23(2):93–8. doi: 10.1038/s41435-022-00169-5 (PMC9012157; doi:10.1038/s41435-022-00169-5)

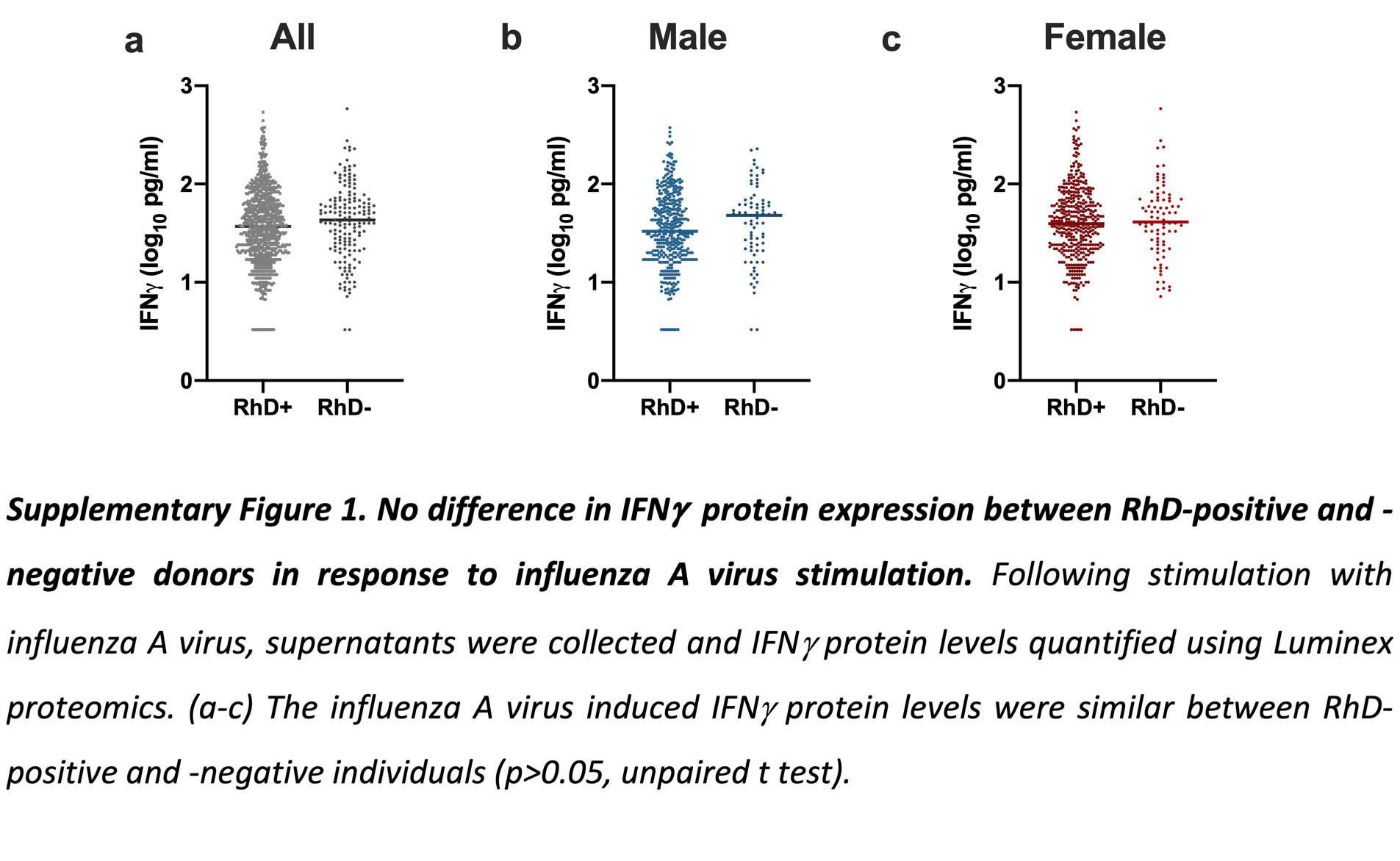

Supplement: Supplementary file 1 — Supplementary Figure 1 [file 41435_2022_169_MOESM1_ESM.tif]
